# Supplementary figures and images for: The unconventional kinesin Kif26a is required for the guidance of major axon tracts in the developing mouse brain
Source: bioRxiv. 2026 May 21:2026.05.20.726728. Preprint. [Version 1] doi: 10.64898/2026.05.20.726728 (PMC13228590; doi:10.64898/2026.05.20.726728)

# Figure 3 supplement 1

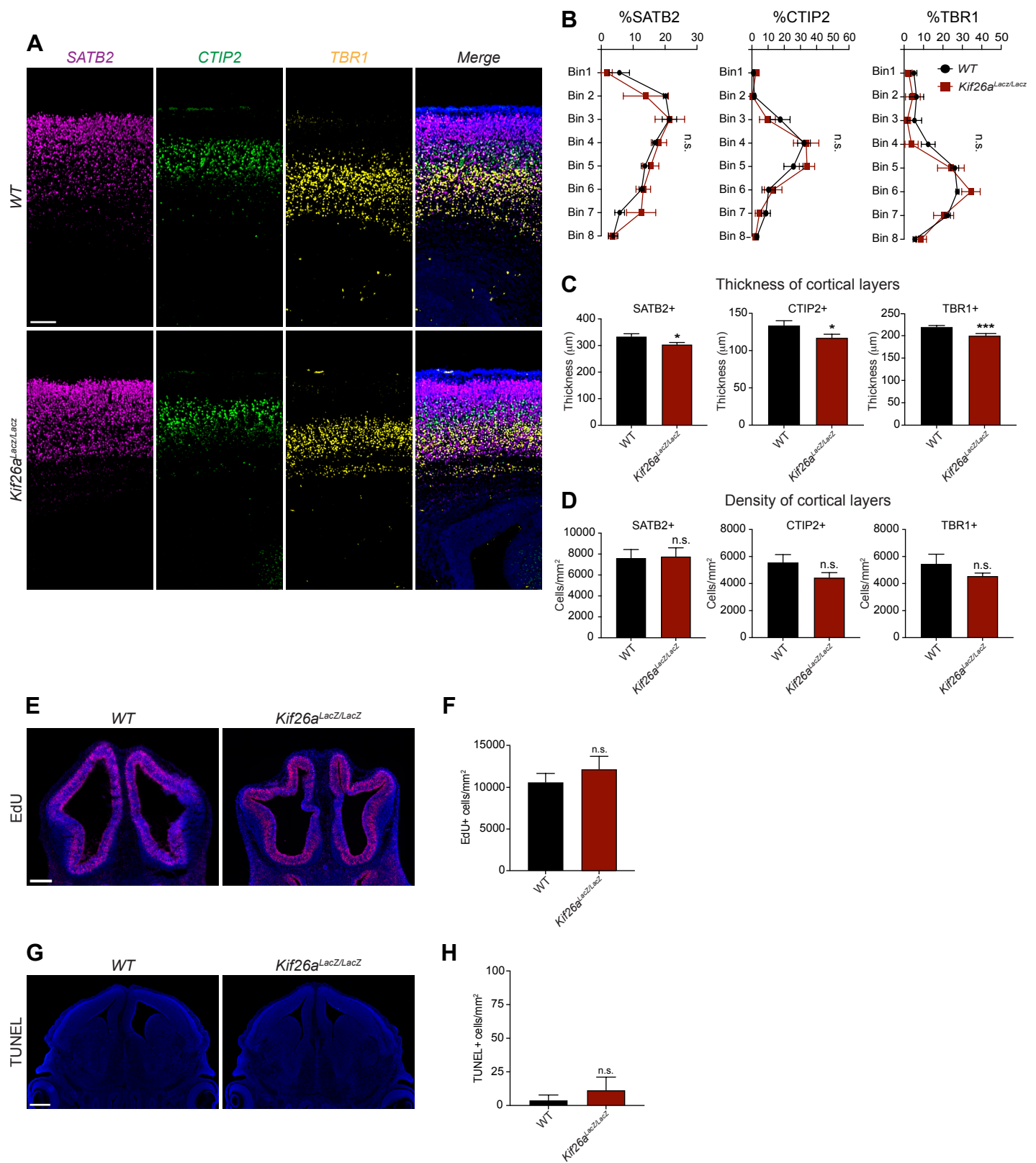

Supplement: Supplement 1 — Figure 3 supplement 1 Analysis of cortical layering, cell proliferation and apoptosis in the Kif26aLacZ/LacZ brain (A) E18.5 cortical sections were immunostained with anti-SATB2 (magenta), -CTIP2 (green), and -TBR1 (yellow) antibodies to mark the superficial, intermediate, and deep cortical layers, respectively. Sections were counterstained with DAPI (blue). Scale bar: 100 μm. (B) Distribution of SATB2+, CTIP2+ and TBR1+ cells across the thickness of WT and Kif26aLacZ/LacZ cortices. The region encompassing the marginal zone to the subplate was virtually divided into 8 bins, and the percentage of cells positive for each marker was quantified for each bin. (C) Quantification of the thickness of SATB2+, CTIP2+, and TBR1+ layers in WT and Kif26aLacZ/LacZ brains. (D) Quantification of the density of SATB2+, CTIP2+, and TBR1+ neurons within the respective layers of the WT and Kif26aLacZ/LacZ brains. (E) Coronal sections of E12.5 WT and Kif26aLacZ/LacZ brains showing the incorporation of EdU (red) as a proxy of cell proliferation. EdU was injected intraperitoneally into pregnant dams carrying E12.5 embryos. Embryos were collected 4 hr post injection. Sections were counterstained with Hoechst 33342 (blue). Scale bar: 250 μm. (F) Quantification of EdU incorporation (number of EdU+ cells per mm2) in the cortex of WT and Kif26aLacZ/LacZ brains. (G) Coronal sections of E14.5 WT and Kif26aLacZ/LacZ brains analyzed for apoptosis by TUNEL assay. Apoptotic cells are shown in green. Sections were counterstained with Hoechst 33342 (blue). Scale bar: 250 μm. (H) Quantification of apoptosis (number of TUNEL+ cells per mm2) in WT and Kif26aLacZ/LacZ brains. Data are represented as mean +SEM of the quantified phenotypes from 3 independent embryos for each genotype. t-Test (unpaired) was performed to determine statistical significance of mutant vs. WT. ***, p < 0.001; *, p < 0.05; n.s., not significant. [file NIHPP2026.05.20.726728v1-supplement-1.pdf]
